# Linking Environmental Drivers to Infectious Diseases: The European Environment and Epidemiology Network

**DOI:** 10.1371/journal.pntd.0002323

**Published:** 2013-07-25

**Authors:** Jan C. Semenza, Bertrand Sudre, Tolu Oni, Jonathan E. Suk, Johan Giesecke

**Affiliations:** Office of the Chief Scientist, European Centre for Disease Prevention and Control (ECDC), Stockholm, Sweden

Europe is increasingly at risk of the emergence of tropical diseases that are more commonly associated with warmer climates [1]. In 2010, Southeastern Europe witnessed an upsurge in West Nile fever cases that was strongly associated with excessive heat waves that had occurred in the preceding weeks [2]. France sustained its first autochthonous dengue transmission in the summer of 2010, and Madeira is currently experiencing the first persistent dengue transmission in Europe since 1920 [3]. In addition, the first outbreak of chikungunya outside the tropics occurred in 2007 in Italy, with subsequent autochthonous cases in France and Croatia in 2010 [4], and since 2009, Greece has experienced a resurgence of indigenous cases of *Plasmodium vivax* transmission in environmentally and climatically suitable areas [5].

Globalization and environmental change, social and demographic factors, and health system capacities are all significant drivers of infectious diseases [6]. International travel and trade can facilitate the dispersal of vectors and pathogens to new areas that are increasingly climatically and environmentally suitable. Environmental factors, such as agriculture, irrigation, and deforestation, are other important determinants of emerging infectious diseases. Urbanization and urban sprawl have encroached upon agricultural and seminatural areas in Europe, and the trend is expected to continue in many places. One consequence of habitat destruction is the displacement of wildlife, sometimes into urban or abandoned environments, which can in turn affect human exposures to infectious pathogens.

In addition to the above, climate significantly influences the transmission of infectious diseases. Many zoonotic enteric pathogens, including *salmonella*, *cryptosporidium*, VTEC, and *campylobacter*, are known to exhibit seasonal patterns, while climatic conditions including temperature and rainfall influence the transmission of vector-borne diseases ranging from tick-borne encephalitis to dengue [7], [8]. Thus anthropogenic climate change, which has accelerated over the past decade, may lead to shifts in the distribution of infectious diseases [9].

Environmental drivers are often epidemic precursors of disease, meaning that monitoring changes in environmental conditions can guide the anticipation and forecasting of upsurges in infectious disease [10]. At an ecological level, the utility of predictive models has been documented for a number of case studies. For example: deer mice density has been used to predict hantavirus infections [11]; rainfall and temperature have been used as predictors of high-risk areas of plague and hantavirus pulmonary syndrome [12]; sea surface temperature, elevated rainfall, and vegetation index have been used to predict outbreaks of Rift Valley fever [13]; expansion of schistosomiasis in China has been linked to climate change scenarios [14]; climate change models have been used to predict Lyme disease risk and to forecast the emergence of tick-borne infectious disease [15]; and shifts in patterns of transmission of plague and tularemia have been assessed in relation to climate change [16].

A common message from these diverse studies is that merging environmental, remotely sensed, demographic, epidemiological, and other relevant data sets on the basis of common spatiotemporal features allows for the integrated analysis of complex interactions. In turn, identifying long-term trends will build the evidence base for strategic public health action, while identifying short-term events linked to environmental conditions can help improve and accelerate early warning and response capabilities.

The European Centre for Disease Prevention and Control (ECDC) has recognised the strategic importance of such capabilities and has developed an information infrastructure coined the European Environment and Epidemiology (E3) Network, aimed at monitoring environmental conditions related to infectious disease threats [17]. The E3 Geoportal (http://E3geoportal.ecdc.europa.eu/), to be launched in 2013, will support data exchanges and sustained collaborations between EU Member States, researchers, and other interested collaborators (Figure 1). The E3 Network has been specifically designed with the intent of promoting European-wide research into environmental infectious disease epidemiology, leveraging existing European Community investments in this field, and facilitating the exchange of relevant data sets. In addition, the E3 Network also aims to provide technical support for the reporting, monitoring, analysis, and mapping of data and to enhance the analytical capacity of existing resources in Europe. Results could then be disseminated to policy makers, public health practitioners, European Union and international agencies, other governmental sectors, and nongovernmental organisations.

**Figure 1:**
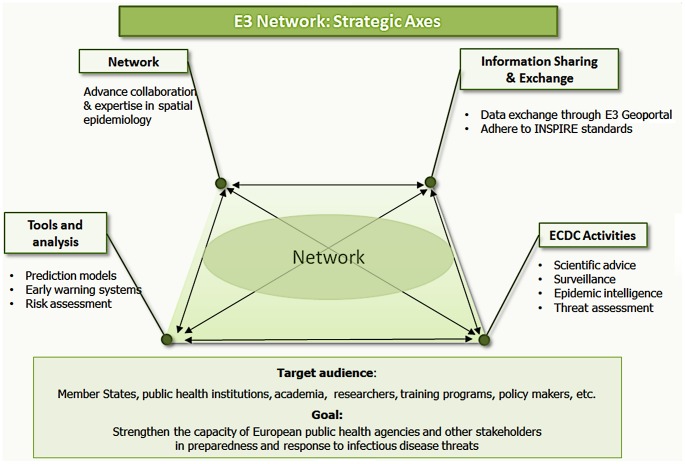
Strategic focus of the E3 Network.

For example, data from the E3 Network have recently been used to delineate areas suitable for malaria transmission in Greece during the resurgence of locally acquired *Plamosdium vivax* [5]. The objective was to define the environmental profile of areas with active transmission cycles between 2009 and 2012 and, based on this, to demarcate other regions at risk for malaria reemergence in Greece. A prediction model was developed with a number of environmental variables, including temperature (day- and nighttime Land Surface Temperature (LST)), vegetation seasonal variations (Normalized Difference Vegetation Index (NDVI)), altitude, land cover categories, and demographic indicators. These variables predicted suitability of areas for persistent malaria transmission characterized by low elevation, elevated temperatures, and intensive, year round irrigated agriculture with complex cultivation patterns (Figure 2). Such spatial analyses can help guide public health preparedness and response to infectious disease threats by directing surveillance and vector control activities.

**Figure 2:**
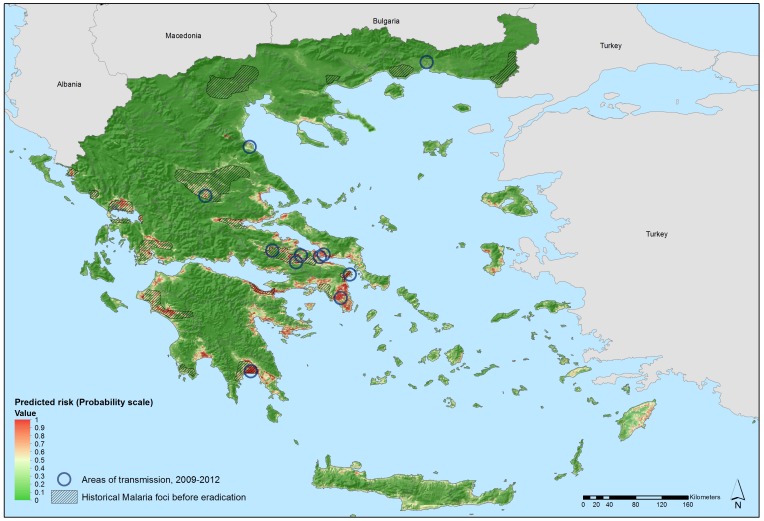
Areas predicted to be environmentally suitable for persistent malaria transmission in Greece, 2013. Note: Map adapted from [5]. Countrywide geo-referenced environmental and climatic data were used from the European Environment and Epidemiology (E3) Network data repository for spatial modeling (nonlinear discriminant analysis). Map illustrates areas predicted to be environmentally suitable for malaria transmission based on *Plamosdium vivax* transmission in Greece from 2009 to 2012. Values from 0 to 0.5 (dark to light green) indicate conditions not favorable for malaria transmission (based on locally acquired cases), as opposed to yellow to dark red areas that delineate conditions increasingly favorable for transmission (values from 0.5 to 1). Areas of historic malaria transmission in Greece refers to the period prior to the elimination in 1974, as a result of a national malaria elimination effort between 1946 and 1960.

## The E3 Geoportal

A number of advances in spatial epidemiology have proven to be instrumental for the establishment of the E3 Network. These include the creation of a wide array of eco-climatic and ecological spatial data archives from a number of sources including the EU FP6 EDEN project that ECDC has acquired, evaluated, and integrated into the E3 Network. A geospatial data repository has been built by ECDC with the capability to store, manage, and serve spatial data layers (e.g., geocoded disease data referenced to a geographic location) as well as other data types. As a central reference point, the E3 data repository will serve data to all E3 Network applications as appropriate and will also supply advanced data querying and analysis functionalities to these applications.

ECDC has created the E3 Geoportal (http://E3geoportal.ecdc.europa.eu/) as a (spatial) data dissemination platform to facilitate the search and discovery of E3 Network resources, and to enable the upload, download, and exchange of data (Table 1). The E3 Geoportal will facilitate the opening up and sharing of all types of information objects that are gathered under the project in a manner that complies with INSPIRE directives (INSPIRE is an EU initiative to assure that spatial data are compatible and usable: http://inspire.jrc.ec.europa.eu/) for providing spatial information services in Europe.

**Table 1:** Environmental and socioeconomic data currently available at the E3 Geoportal, 2013.

**Environmental data**	
Climate change datasets	e.g., WorldClim maximum temperature
Land cover information	e.g., Corine land cover, 2000 EU
Meteorological data	e.g., MODIS snow cover
Vegetation	e.g., Global Lake and Wetlands Database
Hydrology	e.g., Global Lakes and Wetlands Database
Soil data	e.g., Soil Water Index moisture
Wind data	e.g., Wind speed
**Socioeconomic data**	
Population	e.g., Distance weighted population proximity index
Economic	e.g., Country-level GDP
Education	e.g., Pupil/teacher ratio in primary education
Healthcare and hospitals	e.g., Life expectancies for males and females
Transport networks and statistics	e.g., Total length of motorways
Migrant populations	e.g., UNdata net migration rate
Demographic profiles	e.g., Population and living conditions in Urban Audit cities, core city
Agriculture and livestock	e.g., UN FAO crop and livestock production current data and projections

Source: http://E3geoportal.ecdc.europa.eu/.

Four key functionalities include: keyword and browse tree search, map preview, metadata information, and data downloading and uploading. The web portal will serve as the outreach tool for promoting collaboration and building a critical mass of individual, organisational, and consortium partners that will form the network of stakeholders. The E3 Network stakeholders will originate from the following groups: ECDC staff, E3 Network environmental data providers and partners (EU organizations and EU-funded research projects), Member States providing epidemiological data, the WHO (providing epidemiological data), research organizations, public health agencies, and others.

The next steps for the E3 Network include further developing analytical capabilities. This involves exploring the possibilities of enriching the E3 Network service with easy-to-use tools that can simplify and automate some complex analytical processes and enable users to interact with pre-set data layers and obtain desired results. It is envisaged that some utilities developed by external partners of the E3 Network will be converged and harmonised under this exercise.

## Conclusion

One of the key pillars of the recently launched EU Strategy on Adaptation to Climate Change is better informed decision-making (http://ec.europa.eu/clima/policies/adaptation/what/index_en.htm). In the area of infectious disease, it is increasingly acknowledged that an ecological understanding of disease transmission is essential for proactive public health preparedness [17]. With the E3 Network, ECDC is in the process of developing twenty-first century surveillance by monitoring environmental precursors of disease. The E3 Network is a novel surveillance system designed to link environmental data with epidemiological data. The goal of the E3 Network is early detection of, and rapid response to, shifting infectious disease burdens, particularly those of diseases currently associated with the tropics. By monitoring environmental drivers of disease during periods of global environmental change, this data system can supply crucial information and analyses for forecasting, and thereby assist in predicting changing patterns of infectious disease burden. The Network can significantly enhance preparedness and accelerate the public health response to emerging infectious diseases, thereby helping contain human and economic costs, particularly in resource-strapped regions.
